# Altered Thymic Function during Interferon Therapy in HCV-Infected Patients

**DOI:** 10.1371/journal.pone.0034326

**Published:** 2012-04-16

**Authors:** Stephanie Beq, Sandra Rozlan, Sandy Pelletier, Bernard Willems, Julie Bruneau, Jean-Daniel Lelievre, Yves Levy, Naglaa H. Shoukry, Rémi Cheynier

**Affiliations:** 1 Département de Virologie, Institut Pasteur, Paris, France; 2 Centre de Recherche du Centre Hospitalier de l'Université de Montréal (CRCHUM), Hôpital St-Luc, Montréal, Québec, Canada; 3 Département de microbiologie et immunologie, Université de Montréal, Montréal, Québec, Canada; 4 Département de médecine, Université de Montréal, Montréal, Québec, Canada; 5 Département de médecine familiale, Université de Montréal, Montréal, Québec, Canada; 6 Assistance Publique-Hôpitaux de Paris (AP-HP), Groupe Henri-Mondor Albert-Chenevier, Immunologie Clinique, INSERM, U955, Université Paris 12, Créteil, France; 7 Inserm U1016, Département Immunologie-Hématologie, Institut Cochin, Paris, France; 8 CNRS, UMR 8104, Paris, France; 9 Faculté de Médecine René Descartes, Université Paris Descartes, UMR-S 8104, Paris, France; Faculty of Medicine Tel Aviv University, Israel

## Abstract

Interferon alpha (IFNα) therapy, despite good efficacy in curing HCV infection, leads to major side effects, in particular inducement of a strong peripheral T-cell lymphocytopenia. We here analyze the early consequences of IFNα therapy on both thymic function and peripheral T-cell homeostasis in patients in the acute or chronic phase of HCV-infection as well as in HIV/HCV co-infected patients. The evolution of T-cell subsets and T-cell homeostasis were estimated by flow cytometry while thymic function was measured through quantification of T-cell receptor excision circles (TREC) and estimation of intrathymic precursor T-cell proliferation during the first four months following the initiation of IFNα therapy. Beginning with the first month of therapy, a profound lymphocytopenia was observed for all T-cell subsets, including naïve T-cells and recent thymic emigrants (RTE), associated with inhibition of intrathymic precursor T-cell proliferation. Interleukin (IL)-7 plasma concentration rapidly dropped while lymphocytopenia progressed. This was neither a consequence of higher consumption of the cytokine nor due to its neutralization by soluble CD127. Decrease in IL-7 plasma concentration under IFNα therapy correlated with the decline in HCV viral load, thymic activity and RTE concentration in blood. These data demonstrate that IFNα-based therapy rapidly impacts on thymopoiesis and, consequently, perturbs T-cell homeostasis. Such a side effect might be detrimental for the continuation of IFNα therapy and may lead to an increased level of infectious risk, in particular in HIV/HCV co-infected patients. Altogether, this study suggests the therapeutic potential of IL-7 in the maintenance of peripheral T-cell homeostasis in IFNα-treated patients.

## Introduction

The hepatitis C virus (HCV) causes persistent infection in approximately two thirds of cases leading to chronic liver disease, liver failure, and, eventually, hepatocellular carcinoma in a substantial proportion of infected individuals. The most common therapy for chronic hepatitis C consists of pegylated interferon-α (IFNα) and ribavirin administration which results in viral clearance in 43–46% (genotype 1) to 80%, (genotype 3) of treated patients [1]. Interferon will continue to be a major component of new direct acting antivirals for treatment of HCV [2].

IFNα is produced in large amounts during the acute phase of many viral infections [3], [4], [5], [6]. Direct activation of interferon-stimulated genes enhances naïve T-cell survival through increased Bcl-2 and reduced Bax activation [7] and contributes to clonal expansion of antigen-specific T-cells [8]. Recent data suggest that early therapeutic intervention with pegylated IFNα rescues polyfunctional memory T-cells expressing high levels of the IL-7 receptor alpha chain (CD127) and Bcl-2, allowing a higher rate of sustained viral response [9]. However, despite good efficacy, IFNα-based therapies lead to sustained anemia, thrombocytopenia, neutropenia and lymphocytopenia [10], [11], [12], [13], [14]. Moreover, pegylated IFNα therapy enhances the risk of infection in older HCV-infected patients and HIV-infected individuals, independently from neutropenia [15], [16], [17].

The mechanisms of action of IFNα include inhibition of different hematopoietic growth factors [18], [19], possibly affecting lymphoid differentiation at an early stage [20], and modifications in cell homing [12], [21], [22]. The mechanisms involved in IFNα therapy-associated leukocyte depletion remain poorly understood.

Others and we have documented a strong reduction in the ability of HIV-infected patients to sustain thymic production as a direct consequence of a drop in intrathymic precursor T-cell proliferation [23], [24], [25]. Similar thymic impact was also seen during early SIV-infection in the rhesus macaque model [26]. The capacity of the thymus to produce recent thymic emigrants (RTEs) is, in large part, dependent on thymocyte proliferation [27]. Indeed, extensive thymocyte proliferation occurs between T-cell receptor beta (TCRB) and alpha (TCRA) chain rearrangements. The extent of this proliferation directly correlates with thymic export [28]. The extent of cell proliferation in the thymus can be measured in patients through estimating, in peripheral blood cells, the ratio (sj/βTREC ratio) between the frequency of signal joint T-cell receptor excision circles (sjTREC), produced during the excision of the TCRδ locus prior to TCRα chain rearrangement, and that of DβJβTREC T-cell receptor excision circles (TRECs) produced during TCRBD to TCRBJ rearrangement [29]. These by-products of TCR rearrangement processes are generated by the circularization of the chromosomal DNA excised during TCR rearrangements respectively occurring at the DN3 (DβJβTREC) and DP (sjTREC) stages of differentiation. Due to the fact that TRECs do not replicate during mitosis, increased proliferation between DN3 and DP leads to the reduction of DβJβTREC frequency in RTEs as compared to sjTREC frequency and consequently to an increase of the sj/βTREC ratio [23]. The correlation between initial plasma IFNα levels and the speed of thymic dysfunction observed during HIV primary infection suggested that IFNα, produced as part of the innate immune response to infection, participates in the impairment of thymopoiesis. However, no direct evidence of the relationship between IFNα production and thymic dysfunction was provided by these studies. In contrast, Arizcorreta and colleagues showed that IFNα and ribavirin therapy induces a substantial reduction of circulating sjTRECs, in HIV/HCV co-infected patients, accompanied by sustained naïve CD4^+^ T-cell defect, suggesting thymic dysfunction [10]. Similarly, in the SIV-infected rhesus macaque model, we showed that IFNα therapy induced a strong decrease of circulating RTE numbers as defined either by sjTREC frequency and numbers or by CD31^hi^ expression on naïve T-cells [30]. Interestingly, in these animals, recombinant interleukin (IL)-7 therapy more than abrogated the deleterious effects of IFNα therapy [30].

IL-7 is a key cytokine implicated at various levels of thymocytes differentiation. It allows cell survival during the rearrangement processes, and is implicated in the extensive thymocyte proliferation, in particular in intermediate single positive (ISP) and early DP cells [31], [32], [33], [34]. This proliferation participates in the development of naïve T-cell diversity [35]. While up regulated by IFNα [36], [37], the cyclin-dependent kinase inhibitor P27/Kip1 is negatively regulated by IL-7 [38], allowing ISP and early DP thymocytes to proliferate. Moreover, IFNα also inhibit IL-7 dependent proliferation through down modulation of the common γ chain, that participates, together with CD127 to the IL-7 receptor [39]. We here investigated the early impact of IFNα therapy on thymic function and naïve T-cell homeostasis in both HCV-infected and HIV/HCV co-infected patients who started IFNα therapy.

## Results

### IFNα treatment alters circulating naïve T-cell subsets

**Figure 1:**
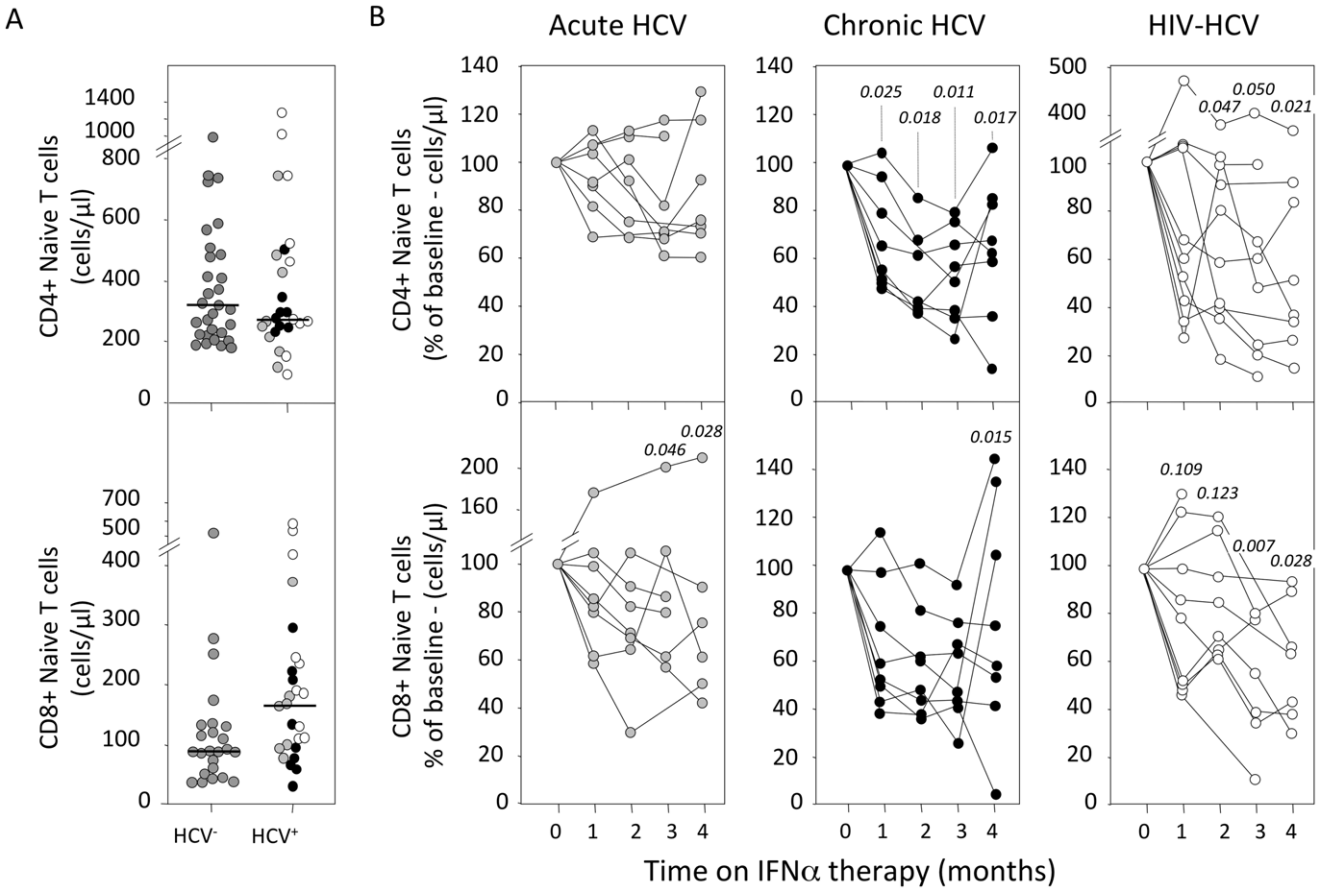
IFNα therapy leads to naïve T-cell lymphocytopenia. (A) CD4^+^ (top panel) and CD8^+^ (bottom panel) naïve T-cell counts were quantified in peripheral blood cells from acutely HCV-infected (light grey symbols), chronically HCV-infected (black symbols) and HIV/HCV co-infected (white symbols) patients at study entry, as compared to healthy donors (HCV-, dark grey symbols). (B) Evolution of CD4^+^ (top panels) and CD8^+^ (bottom panels) naïve T-cell counts during the first 4 months of IFNα therapy in acutely HCV-infected (left panels), chronically HCV-infected (central panels) and HIV/HCV co-infected (right panels) patients. Each line represents data from an individual patient. Statistical significances of the differences to baseline values (time 0), calculated on the absolute naïve T-cell counts in each individual sample, (Wilcoxon matched-pairs signed-ranks test) are shown on top. The horizontal bars represent median values.

We first evaluated the evolution of naïve T-cell subsets in three groups of HCV infected individuals: 1) Acute HCV infection (n = 8), defined as <6 months post estimated date of infection; 2) chronic HCV infection (n = 8), defined as >6 months post estimated date of infection; and 3) HIV/HCV co-infected individuals (n = 10). In all groups, patients were enrolled at the beginning of IFNα therapy and were followed for a total of 4 months. While, for any group of patient's, naïve CD4+ and CD8+ T-cell counts were not significantly different from healthy individuals (figure 1A), as early as one month following treatment initiation, naïve CD4+ T-cell counts were significantly reduced in chronically HCV-infected patients (39%, 58%, 46% and 35% decrease at M1, M2, M3 and M4 respectively; p≤0.025; Figure 1B, top central panel). A similar trend was also observed in the CD8 compartment (40%, 39%, 33% and 33% decrease; Figure 1B, bottom central panel). A comparable effect was also observed in most co-infected patients (mean cell count declines were 19%, 32%, 52% and 43% at M1, M2, M3 and M4 in the CD4+ T-cell compartment and 9%, 21%, 41% and 42% in CD8+ T-cell subset; p≤0.05 by M2–M3; Figure 1B, right panels). In contrast, naïve T-cell counts were only barely affected in acutely-HCV infected patients under IFNα therapy (Figure 1B, left panels). Similarly, central memory CD4+ T-cells (CD45 RA- CCR7+; TCM) demonstrated 38% and 28% decrease in HCV and HIV/HCV patients respectively (59% and 60% in CD8+ TCM) while effector memory (CD45RA− CCR7–; TEM) CD4+ T-cell counts declined by 45% and 10% in the same groups (61% and 65% in CD8+ TEM) (Figure S1).

**Figure 2:**
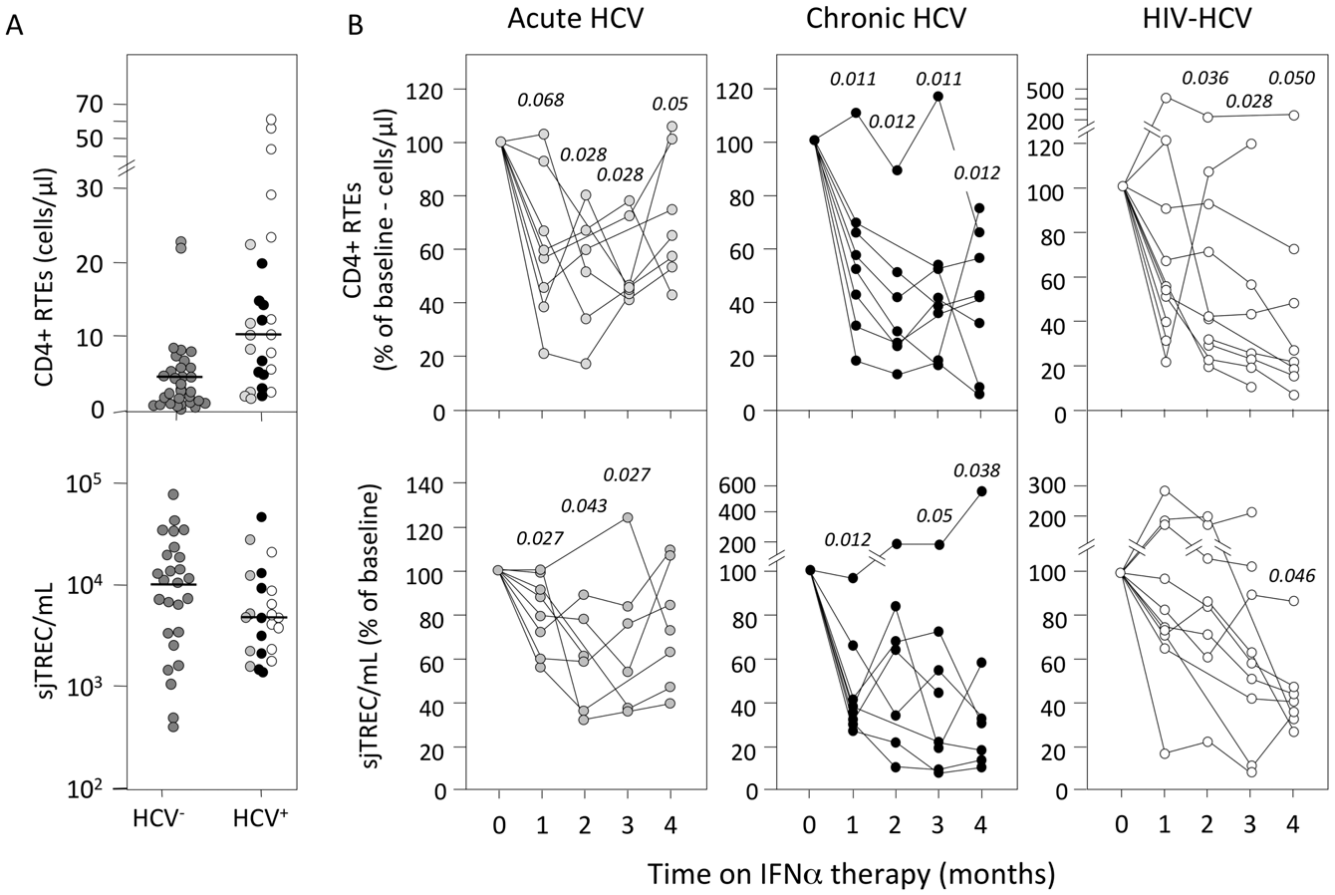
IFNα therapy reduces recent thymic emigrant blood counts. (A) Recent thymic emigrant counts (RTE/μl, top panel) and sjTREC concentration (TRECs/ml, bottom panel) were quantified in peripheral blood cells from acutely HCV-infected (light grey symbols), chronically HCV-infected (black symbols) and HIV/HCV co-infected (white symbols) patients at study entry, as compared to healthy donors (HCV^−^, dark grey symbols). (B) Evolution of (RTE/μl, top panels) and sjTREC concentration (TRECs/ml, bottom panels) during the first 4 months of IFNα therapy in acutely HCV-infected (left panels), chronically HCV-infected (central panels) and HIV/HCV co-infected (right panels) patients. Each line represents data from an individual patient. Statistical significances of the differences to baseline values (time 0), calculated on the absolute RTE CD4^+^ T-cell counts and sjTREC levels in each individual sample (Wilcoxon matched-pairs signed-ranks test) are shown on top. The horizontal bars represent median values.

Within CD4+ naïve T-cells, RTEs can be identified by their higher expression of the platelet endothelial cell adhesion molecule-1 (PCAM-1 or CD31) [40]. While the number of RTEs was similar in HCV-infected patients at study entry and healthy individuals (Figure 2A, top panel), the proportion of CD31hi cells in naïve CD4+ (CD45RA+ CCR7+) T-cells was significantly reduced by M1 in acutely HCV-infected patients (p<0.05 at all time points). Together with the decline in naïve T-cell counts, this translated into reduced numbers of circulating RTE (p≤0.05 by M2; Figure 2B, top left panel). Similarly, chronically HCV-infected patients demonstrated lower absolute numbers of CD31hi naïve T-cells by M1 (p≤0.012; Figure 2B, central panel). In the co-infected patients group, despite more limited variations in the percentage of RTEs in naïve T-cells (p≤0.05 at M1 and M4), the absolute RTE counts also declined with time under therapy (p≤0.05 at M2, M3 and M4; Figure 2B, right panel).

RTE concentration in blood can also be estimated through quantification of the sjTREC content (sjTREC/mL, Figure 2, bottom panels). sjTREC content was in the range of age matched healthy individuals at baseline (figure 2A) but declined significantly in both subgroups of HCV-infected patients by one month following initiation of IFNα therapy (median = 5034, 4104, 2980, 2805 and 3076 sjTREC/mL at M0, M1, M2, M3 and M4 respectively in acutely HCV-infected patients; p<0.05 and median = 3879, 1895, 2018, 1511 and 1040 sjTREC/mL at M0, M1, M2, M3 and M4 respectively in chronically HCV-infected patients; p<0.05; Figure 2B, left and central bottom panels). In contrast, HIV/HCV infected patients demonstrated more stable sjTREC/mL values that eventually declined at M4 (median  = 4192, 5215, 4420, 3871 and 1597 sjTREC/mL at M0, M1, M2, M3 and M4 respectively (p = 0.046 at M4); Figure 2B, right bottom panel).

These data demonstrate that, as early as one month following treatment initiation, IFNα induces stronger alterations of naïve T-cell subsets, and more specifically in the RTE compartment than in any other T-cell subset, suggesting a specific effect on thymopoiesis. We thus analyzed the evolution of intrathymic precursor T-cell proliferation, peripheral T-cell cycling, IL-7 plasma concentration and IL-7 receptor alpha chain (CD127) expression, different factors affecting naive T-cell homeostasis.

### IFNα therapy affects thymic function

**Figure 3:**
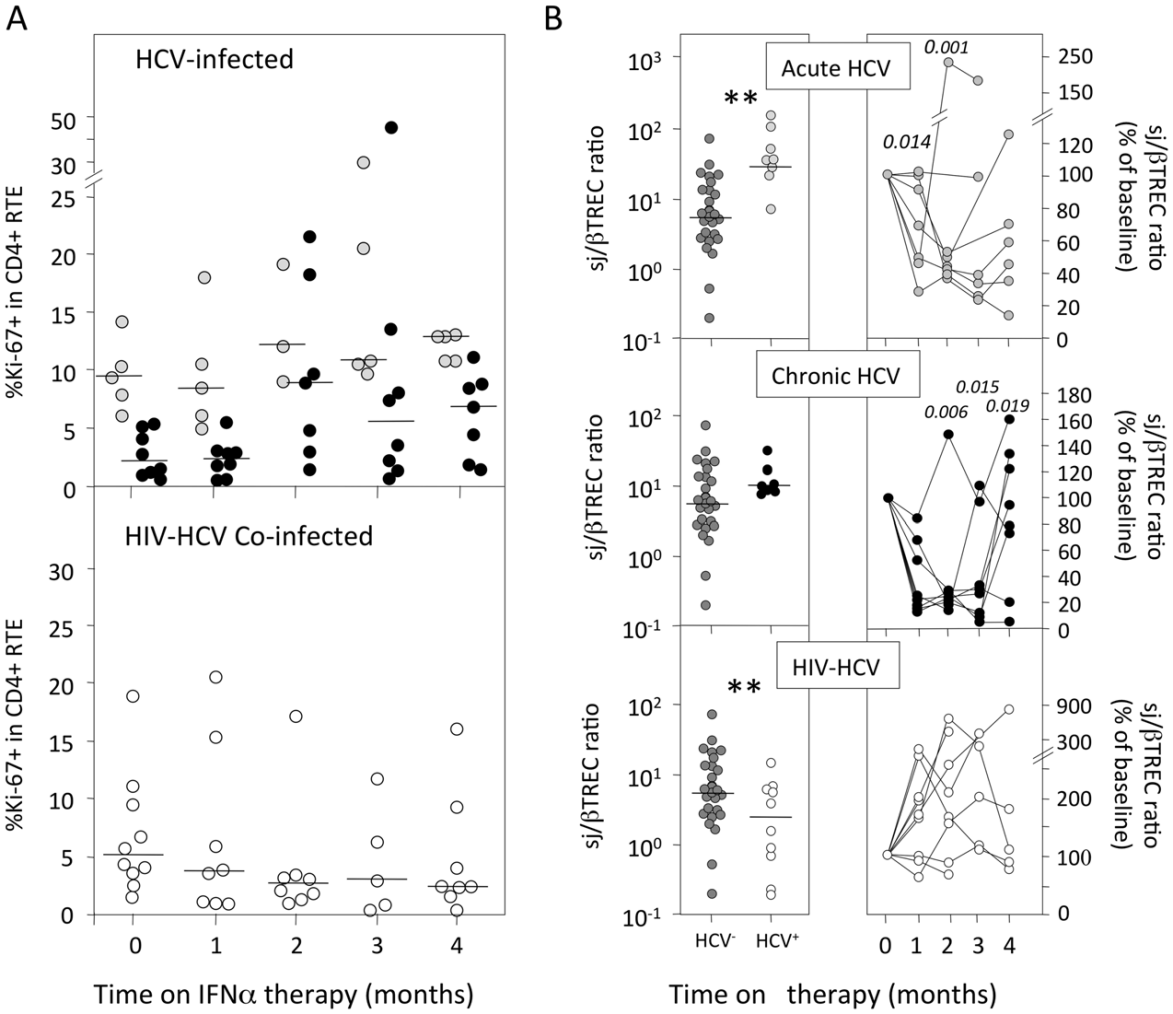
IFNα therapy leads to major impairment of thymic function. (A) The frequency of Ki-67 expressing cells in the CD4^+^ RTE subset (CD31^hi^ naïve T-cells) was measured in acutely HCV-infected (grey symbols, top panel), chronically HCV-infected (white symbols, top panel) and HIV/HCV co-infected (bottom panel) patients (central panels) and HIV/HCV co-infected (right panels) patients. Each line represents data from an individual patient. Statistical significances of the differences to baseline values (time 0), calculated on the absolute sj/βTREC ratio in each individual sample (Wilcoxon matched-pairs signed-ranks test) are shown on top. The horizontal bars represent median values.

Despite differences between the 3 groups at study entry, RTE cycling rate, as estimated through measurement of Ki-67 expression, did not change significantly during the follow-up period (Figure 3A). These data demonstrate that the observed changes in sjTREC frequencies were not a consequence of variations of RTE proliferation during IFNα therapy but more probably due to reduced thymic production.

We thus estimated thymic output through quantification of the sj/βTREC ratio in all groups of patients (Figure 3B). The sj/βTREC ratio estimates the extent of thymocyte proliferation between TCRB rearrangement and the excision of the T-cell receptor delta (TCRD) locus [23]. This parameter directly reflects the extent of thymic production and, contrarily to sjTREC values, is independent from peripheral RTE proliferation or survival capacity [28]. The sj/βTREC ratio was already low in HIV-infected patients (p<0.005 as compared to healthy control donors; Figure 3B bottom left panel) and did not evolve further under IFNα therapy in co-infected patients (Figure 3B, bottom right panel). In contrast, acutely HCV-infected patients demonstrated higher than normal sj/βTREC ratio at baseline (p<0.05 as compared to aged matched healthy controls), showed a significant reduction in sj/βTREC ratio at M1 (p = 0.014) and M2 (p = 0.001; Figure 3B, top panel). Finally, a similar decline in the sj/βTREC ratio was observed during IFNα therapy in chronically HCV-infected patients (p<0.02 at M1, M2 and M3; Figure 3B, central panel).

### Reduction of IL-7 plasma levels under IFNα treatment

**Figure 4:**
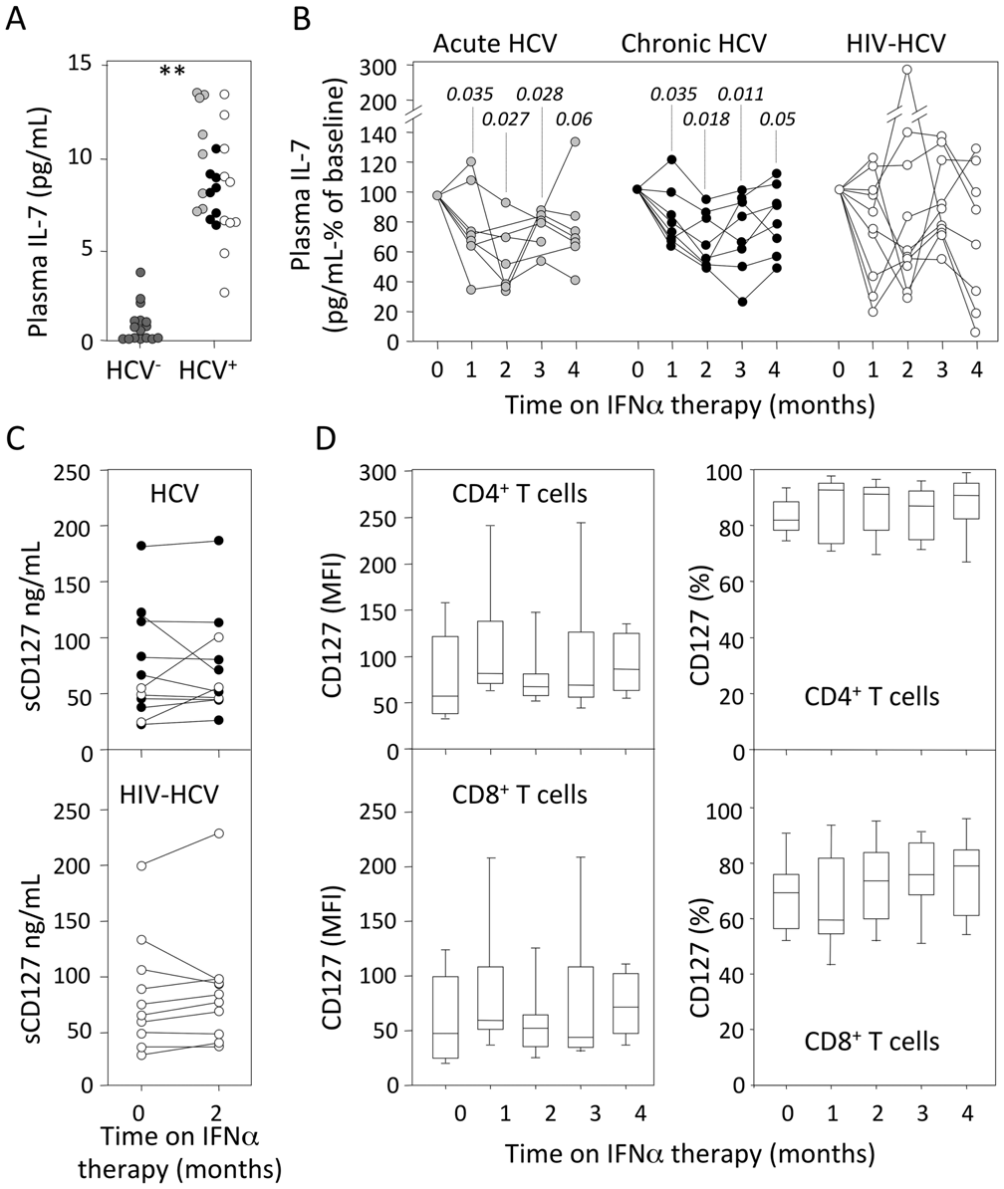
IFNα therapy leads to reduction in IL-7 plasma concentration. (A) IL-7 plasma levels were quantified in peripheral blood cells from acutely HCV-infected (light grey symbols), chronically HCV-infected (black symbols) and HIV/HCV co-infected (white symbols) patients at study entry, as compared to healthy donors (HCV-, dark grey symbols). **: p<0.001 for any HCV-infected patients group. (B) Evolution of plasma IL-7 levels over the first 4 months of IFNα therapy in acutely HCV-infected (left panels), chronically HCV-infected (central panels) and HIV/HCV co-infected (right panels) patients. Each line represents an individual patient. Statistical significances of the differences to baseline values (time 0), calculated on the absolute IL-7 plasma levels in each individual sample (Wilcoxon matched-pairs signed-ranks test) are shown on top. (C) Soluble CD127 was quantified in plasma from acutely HCV-infected (white symbols, top panel), chronically HCV-infected (black symbols, top panel) and HIV/HCV co-infected (bottom panel) patients at baseline (0) and M2. (D) CD127 expression was measured on circulating CD4^+^ (top panel) and CD8^+^ (bottom panel) and expressed as mean fluorescence intensity (left panels) and percentages of positive cells (right panels) over the 4 first months of IFNα therapy.

Precursor T-cell proliferation in the thymus is, at least in part, dependent upon IL-7. We thus quantified plasma IL-7 concentration in all groups of patients. At study entry, HCV- and HIV/HCV-infected patients presented with elevated plasma IL-7 (median = 10.3 pg/mL, range (6.7–12.9) in acutely HCV-infected patients; 8.3 pg/mL (6.3–10.5) in chronic HCV-infected patients and 7.15 pg/mL (4.3–13.5) in co-infected subjects), as compared to that observed in healthy control individuals (p<0.001 for any patients' group; Figure 4A). Surprisingly, while lymphocytopenia established, IL-7 plasma concentrations significantly decreased in both groups of HCV-infected patients (30, 54, 18 and 29% decrease at M1 to M4 in acute infection, p<0.05; 25, 46, 26 and 16% decrease at M1 to M4 in chronic infection, p<0.05; Figure 4B left and central panels). In contrast, IL-7 plasma levels did not significantly evolve in co-infected individuals during the first month of IFNα therapy (Figure 4B right panel). Only patients with the highest IL-7 plasma levels showed a reduction in the concentration of this cytokine.

Decreased plasma IL-7 concentrations could be a consequence of reduced IL-7 production, increased consumption by T-cells or sequestration by soluble IL-7 receptor (sCD127). In both HCV-infected and HIV/HCV co-infected patients, neither sCD127 plasma concentration (Figure 4C) nor CD127 expression by CD4+ or CD8+ T-cells (Figure 4D) significantly changed during IFNα therapy.

### Evolution of Thymic function parallels IL-7 plasma levels

Considering the variations in all the parameters we used to evaluate thymic function, we then sought to evaluate the impact of changes in IL-7 plasma levels on de novo production from the thymus and on the number of both sjTREC and circulating CD4+ RTEs.

**Figure 5:**
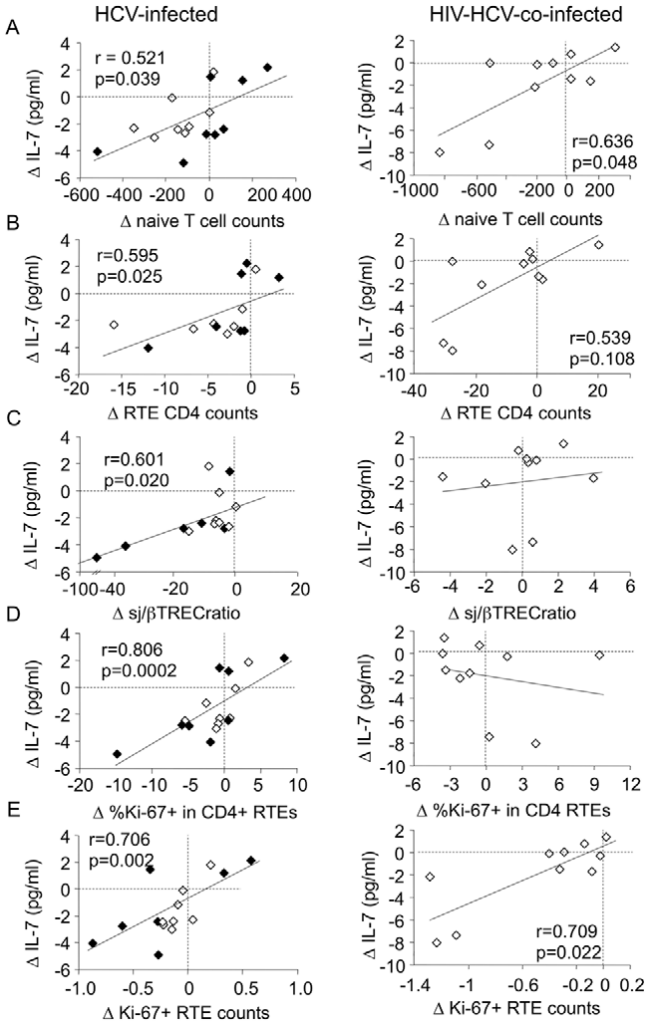
Variations in IL-7 plasma levels correlate with evolution of RTE **production.** Correlations. between variations in IL-7 plasma levels (ΔIL-7) and either variations in (A) total (CD4^+^ + CD8^+^) naïve T-cell counts (Δnaïve T-cell counts), (B) RTE defined as CD31^hi^ naïve CD4^+^ T-cells (ΔRTE CD4 counts), (C) the sj/βTREC ratio (Δsj/βTREC ratio), (D) the frequency of Ki-67^+^ cells in the RTE CD4^+^ T-cell subset (Δ%Ki-67^+^ in CD4^+^ RTEs) or (E) the number of circulating Ki-67^+^CD4^+^ RTEs (ΔKi-67^+^ RTE counts) between study entry and month 1 of therapy were calculated for acutely (black symbols) and chronically (white symbols) HCV-infected patients (left panels) and HIV/HCV co-infected patients (right panels). Correlation coefficients (Spearman's r) and the associated probabilities (p) are shown.

In a majority of patients, IL-7 plasma level, sj/βTREC ratio, sjTREC/ml and blood RTE concentration fluctuated in parallel (Figure S2). Variation of IL-7 plasma concentration (ΔIL-7) during the first month of therapy correlated with variations in naïve T-cell counts (CD4+ + CD8+; ΔNaïve T-cell counts) and RTE CD4+ T-cell counts (ΔRTE T-cell counts) in both HCV (r = 0.521, p = 0.039 and r = 0.595, p = 0.025; Figure 5A and 5B, left panels) and, to a lesser extent, HIV/HCV co-infected patients (r = 0.636, p = 0.048 and r = 0.539, p = 0.108; Figure 5A and 5B, right panels). Moreover, in HCV-infected patients, ΔIL-7 also correlated with variations in intrathymic precursor T-cell proliferation (Δsj/βTREC ratio; r = 0.601, p = 0.020; Figure 5C).

Variations in plasma IL-7 levels also correlated with changes in the proportions (Δ%Ki-67+ in CD4+RTEs; r = 0.806, p = 0.0002; Figure 5D, left panel) and numbers (ΔKi-67+RTEs; r = 0.706, p = 0.002; Figure 5E, left panel) of cycling RTEs in acute and chronic HCV infected patients and with Δ%Ki-67+RTE counts in co-infected patients (r = 0.709, p = 0.022; Figure 5E, right panel). Overall, IL-7 concentration was associated with reduced thymopoiesis and RTE proliferation, lower consequently leading to limited circulating RTE and naïve T-cell counts. These data strongly suggest that changes in IL-7 plasma levels during IFNα therapy directly impact the homeostasis of RTEs.

## Discussion

We herein demonstrated that IFNα-based therapy leads to major lymphocytopenia in naïve T-cell compartments, in particular in the RTE subset. Several mechanisms could be implicated in the establishment of such a lymphocytopenia [41]. Among these, enhanced apoptosis [42], [43], cell sequestration in lymphoid or non-lymphoid organs [12], [21], [22] and regulation of peripheral T-cell homeostasis [20]. In our study, no major change in cell survival (Bcl-2 expression) or T-cell activation (CD25 and CD69 expression) was observed during the follow-up period (data not shown). Moreover, we did not observe any significant modification in Ki-67 expression in any T-cell subset during the first month of therapy (data not shown and Figure 3). Finally, IFNα-induced T-cell homing, although rapid and massive, is only a transient process [22] suggesting that this mechanism marginally contributes to the observed long lasting lymphocytopenia.

Interestingly, both sjTREC quantification (sjTREC/mL) and intrathymic precursor T-cell proliferation (sj/βTREC ratio) were affected very early on after initiation of therapy (Figures 2B and 3B). While sjTREC frequency and concentration in peripheral blood can be affected by modifications of parameters that impact on peripheral T-cell homeostasis (cycling, survival/apoptosis, homing), the sj/βTREC ratio is a marker of the intrathymic proliferation history of RTEs. Indeed, this parameter is generated by cell proliferation that occurs between TCRβ chain rearrangement and the excision of TCRδ locus. Further cell cycling after TCRα chain rearrangement does not modify the sj/βTREC ratio as both type of TRECs are similarly diluted upon cell proliferation. Accordingly, while exported to the periphery, the sj/βTREC ratio of mature T-cells cannot be modified. Therefore, while the observed decrease in sjTREC concentration (figure 2) can be a consequence of modifications of circulating T-cell homeostasis, the decline of the sj/βTREC ratio observed during the first months of IFNα therapy (figure 3) defines changes in thymocyte proliferation, thus in thymic output [28]. Acutely infected patients demonstrated a higher sj/βTREC ratio at baseline than patients in the chronic phase. However, this group was younger (Median = 31.5 (26–47)) versus Median = 53.5 (37–61)) than the chronic group (p<0.01; data not shown) and demonstrated normal sj/βTREC ratio for their age. Similar evolution of thymic function and circulating T-cell subsets were observed in both groups of HCV-infected patients, irrespective of the development stage of HCV pathology. The lack of effect of IFNα therapy in HIV/HCV co-infected patients might be due to the fact that, as expected for chronically HIV-infected individuals, these patients already had a low thymic function at study entry. The impairment of thymopoiesis in HCV-infected patients under IFNα therapy is reminiscent of that observed during the acute phase of HIV-1 infection [23] which suggested that long term production of IFNα, as part of the anti-HIV innate immune response, may play a role in the observed thymic defect. The correlation between decline in IL-7 plasma levels under IFNα therapy and both thymic dysfunction and reduced T-cell counts, in particular in the naïve and RTE compartments (Figures 5A and 5B), confirms this hypothesis. Finally, in a recent study, we showed that IFNα treatment leads to decreased sjTREC frequency as well as reduced naïve T-cell and RTE counts in SIV-infected rhesus macaques [30]. Such an effect was accompanied by a 30–40% decrease in IL-7 plasma levels in these animals and could be counteracted by injection of recombinant simian IL-7 [30]. One could expect that such an effect of type I IFNs is not restricted to HIV-infection as many viral infections induce IFNα responses and cause transient lymphocytopenia in the infected hosts [3], [4], [5], [6]. Moreover, the IFNα-induced reduction of thymic function and its probable consequences on naïve T-cell diversity may contribute to the higher infectious risk associated with IFNα therapy, in particular observed in older patients [15], [16], [44]. There are multiple sources for circulating IL-7 during viral infections including lymphoid organs, epithelial cells and recently the liver was identified as a major source of IL-7. Moreover, increased plasma IL-7 levels can also be observed during viral infection in non-lymphopenic individuals ([33] and unpublished data), suggesting a role in the development of immune responses. Indeed, this cytokine participates to T-cell homing in various lymphoid and non-lymphoid tissues through stimulation of local chemokine productions [45]. Increased IL-7 plasma levels in lymphopenic individuals is likely due to reduced consumption [46] yet augmented production to counteract lymphopenia cannot be excluded [33]. The recent identification of the liver as an IL-7 producing tissue upon TLR stimulation [47] makes it tempting to speculate that HCV-infection can also, through TLR activation, stimulate IL-7 production by the liver. Indeed, non-lymphopenic HCV-infected patients demonstrate similar IL-7 plasma levels than lymphopenic HIV-infected individuals [33], [48] suggesting that most of the IL-7 production in untreated HCV-infected patients was not linked to circulating T-cell counts. The reduction of IL-7 plasma levels while lymphocytopenia establishes under IFNα therapy, the absence of a correlation between IL-7 plasma levels and CD127 expression and the concomitance of decreases in IL-7 plasma levels and HCV viral load under therapy suggest that viremia might be driving IL-7 production before initiation of therapy. Our data suggest that, before initiation of IFNα therapy, actively replicating HCV leads to the overproduction of IL-7. Subsequent reduction of IL-7 production upon initiation of therapy probably reflects the elimination of IL-7 producing HCV-infected hepatocytes. This sudden reduction of IL-7 plasma levels may lead to diminished thymopoiesis. The fact that IL-7 plasma levels did not reach normal levels when HCV became undetectable may suggest that, after the initial decline that follows the drop in viremia, IL-7 plasma levels were regulated, as in HIV-infected patients [33] and in IFNα-treated SIV-infected rhesus macaques [30], as a consequence of lymphocytopenia through either reduced consumption or increased production in lymphoid organs [49]. Future studies with a longer follow-up period, in particular after the end of IFNα therapy and recovery from lymphocytopenia are required to further elucidate this point.

We herein demonstrated that a substantial reduction in thymic export was observed in HCV-infected patients, during the first months of IFNα therapy. This effect directly paralleled IFNα-induced lymphocytopenia and decreased IL-7 plasma levels, initially high in HCV-infected patients. These data suggest that IL-7 production by the liver, a consequence of active HCV replication, was reduced while patients controlled HCV viremia. Restricted IL-7 plasma levels might, in association with the anti-proliferative effect of IFNα, limit T-cell production in the thymus. Our study highlights the therapeutic potential of IL-7 as a complement to the standard IFNα based treatment to help HCV-infected patients to sustain normal circulating T-cell counts, and restore the diversity of the peripheral T-cell repertoire through its central thymopoietic effect. Restoring the breadth and intensity of T-cell control over the HCV virus might be immediately beneficial for the HIV/HCV co-infected population and offer new promising avenues for chronic HCV in the context of massive drop of HCV viral load after short term treatment with new antiviral compounds that will continue to be administered in combination with IFNα [50].

## Patients and Methods

### Patients characteristics

Sixteen HCV-infected patients (C-1 to C-16) and ten HIV/HCV co-infected patients (I-1 to I-10) naïve to IFNα therapy were enrolled in this study. A summary of the virological and immunological status of patients at baseline is shown in table 1. All the HIV/HCV co-infected patients but one were under HAART with undetectable viremia (<40 HIV copies/mL). Chronically infected patients (C-9 to C-16 and I-1 to I-10) initiated pegylated IFNα/ribavirin treatment (IFNα-2a: Pegasys, 180 μg weekly, Ribavirin: Copegus, 800 mg to 1000 μg daily) and were followed over a 4 months period. Patients included in the acute phase of HCV infection (C-1 to C-8) were treated with pegylated IFNα (IFNα-2a: Pegasys, Roche, 180 μg weekly) [51], [52]. Blood samples were taken monthly on EDTA. Two milliliters of total blood were 2-fold diluted in FCS/20%DMSO frozen at −80°C and conserved in liquid nitrogen. These total blood samples were subsequently used for flow cytometry analyses. Plasma was separated from the remaining eight milliliters and mononuclear cells were purified on Ficoll Hypaque (Eurobio, Courtaboeuf, France) and frozen for further analyses. Patients from the HCV mono-infection group were followed at the Centre de Recherche du CHUM, Hôpital Saint Luc, Montreal, QC, Canada and its collaborators as previously described [9], [53]. Patients from the HIV-HCV groups were followed at the Hôpital Henri Mondor, Creteil, France. Clinical protocols conformed to ethical guidelines of the authors' institutions and the US Department of Health and Human Services' human experimentation guidelines. This study was approved by both the Ethical committee of Centre Hospitalier de l'Université de Montreal (CHUM) and the ethical committee of Hôpital Henri Mondor, Créteil, France. Samples were obtained with the written subjects' informed consent.

**Table 1:** Patients' characteristics.

Patient Code	Gender	Age at Tx start	HCV genotype	HCV stage	Baseline Viral load^a,b^		12 weeks Viral load
C-1	F	33	3a	Acute		2.4E+4^b^	<50^c^
C-2	M-->F	30	1	Acute		2.8E+4^b^	<50^c^
C-3	M	39	2b	Acute		<1000^b^	<50^c^
C-4	M	26	3a	Acute		4.78E+6^b^	<50^c^
C-5	M	26	1a	Acute		<600^a^	<50^c^
C-6	M	31	1b	Acute		<600^a^	<50^c^
C-7	M	46	3a	Acute		1.56E+4^b^	<50^c^
C-8	M	45	1a	Acute		1,21E+06^ b^	<50^c^
C-9	M	36	1b	Chronic		8,50E+06^a^	<50^c^
C-10	M	39	1a	Chronic		1,28E+07^a^	<50^c^
C11	F	46	1a	Chronic		8,26E+06^a^	<50^c^
C-12	M	52	1b	Chronic		2,70E+06^a^	<50^c^
C-13	M	55	1a	Chronic		4,79E+06^a^	4,69E+03^a^
C-14	M	58	1a	Chronic		1,73E+07^a^	<50^c^
C-15	M	61	1a	Chronic		3,70E+05^a^	<50^c^
C-16	M	61	1b	Chronic		3,00E+06^a^	3,47E+04^a^
I-1	M	42	3	Chronic		NA	<12^d^
I-2	M	48	3a	Chronic		1.51E+6	<12^d^
I-3	M	39	3	Chronic		7.6E+5	<12^d^
I-4	M	48	3a	Chronic		6.4E+4	<12^d^
I-6	F	53	2a/2c	Chronic		3.0E+6	<12^d^
I-7	M	43	2a/2c	Chronic		4.3E+6	<12^d^
I-8	M	41	1	Chronic		6.7E+5	2.4E+5
I-9	M	43	1	Chronic		1.8E+6	<12^d^
I-10	M	41	4	Chronic		6.6E+5	1.6E+5
I-11	M	39	1	Chronic		7.7E+5	1.6E+4

aCOBAS Amplicor HCV Monitor test, Version 2.0 (sensitivity 600 IU/ml).

bIn house real time quantitative PCR assay (sensitivity 1000 IU/ml).

cQualitative COBAS AmpliPrep/COBAS Amplicor HCV test, version 2.0 (sensitivity 50 IU/ml).

dAbbott RealTime HCV assay (sensitivity 12 IU/ml).

NA: Not available.

### Immunophenotyping and flow cytometry analysis

FACS analyses were performed on cryopreserved samples. After thawing blood cells were incubated for 15 minutes at 4°C with conjugated monoclonal antibodies (mAbs). For intracellular labeling, cells were permeabilized with the Cytofix/Cytoperm Kit (Becton Dickinson) before incubation with specific mAbs according to the manufacturer's instructions. Samples were then washed, fixed in 2% paraformaldehyde phosphate-buffered saline (PBS/PFA 2%) and acquired using a Cyan cytofluorometer (Dako) and analyzed with FlowJo 8.7 software.

The monoclonal antibodies used in this study were: CD3-pacific blue (PB) (clone UCHT-1; Dako, Trappes, France), CD4-peridin chlorophyll protein-cyanine 5.5 (PerCP-Cy5.5) (clone L200; BD, Le-Pont-de-Claix, France), CD45RA-phycoerythrin (PE) (clone HI100; BD), CCR7-allophycocyanin (APC) (clone 150503; R&D Systems Europe, Lille, France); CD8-phycoerythrin-cyanine 7 (PE-Cy7) (RPA-T8; BD), CD31-biotin (clone WM59; AbDSerotec, Düsseldorf, Germany); Ki-67-fluorescein isothiocyanate (FITC) (clone MIB-1; Dako), Bcl-2-FITC (clone 124; Dako) and strepatavidin-PE-Texas-RED (BD).

### IL-7 plasma quantification

IL-7 was quantified in the plasma using the IL-7 Quantikine HS kit according to the manufacturer's instructions (R&D Systems Europe). Plasma soluble-CD127 quantification Soluble plasma IL-7 receptor (sCD127) quantification was performed as previously described [54].

### TREC quantifications

Parallel quantification of the sjTREC and the 13 DJβTRECs, together with CD3γ gene (used as a housekeeping gene) was performed for each sample using LightCyclerTM technology (Roche Diagnostics) with a technique adapted from [29]. Intrathymic precursor T-cell proliferation was evaluated through calculation of the sj/βTREC ratio as described [23].

### HCV RNA quantification

HCV RNA quantification was performed using an in-house quantitative real-time reverse transcription-PCR assay as previously described [9], COBAS Amplicor HCV Monitor test™, Version 2.0 (sensitivity 600 IU/ml)), qualitative COBAS AmpliPrep/COBAS Amplicor HCV test™, version 2.0 (sensitivity 50 IU/ml) or Abbott RealTime HCV assay ™ (sensitivity 12 IU/ml).

### Statistical analysis

Statistical analyses (Spearmans rank correlations and Wilcoxon matched -paired signed-rank tests) were performed using the Stata/IC 10.0 (Stata corporation, College Station, Tx U.S.A.). Due to the exploratory nature of the study there was no correction for multiple comparisons, and calculated p values are reported herein.

## Supporting Information

Figure S1 **IFNα therapy leads to T-cell lymphopenia in memory compartments.** Evolution of (A) CD4+ TCM (top panels) and CD4+ TEM (bottom panels) T-cell numbers, as well as (B) CD8+ TCM (top panels) and CD8+ TEM T-cell counts (bottom panels), quantified in peripheral blood cells from acutely and chronically HCV-infected (left panels white and grey symbols respectively) and HIV-HCV co-infected (right panels) patients under IFNα therapy. Horizontal bars represent median values. Statistical significance (Wilcoxon matched-pairs signed-ranks test) to baseline values (M0) are shown on top.
(TIF)

Figure S2 **IL-7 plasma level parallels RTE concentration and thymic function.** IL-7 plasma levels (grey squares), RTE (CD31^Hi^ naïve CD4+ T-cell blood counts; open diamonds), thymic function (sj/βTREC ratio; close diamonds) and sjTREC concentrations (sjTREC/μl; grey diamonds) were longitudinally quantified in IFNα-treated HCV and HIV-HCV infected patients over a 4 month period. Representative examples of HCV-infected (A) and HIV-HCV co-infected (B) patients are shown.
(TIF)
