# Supplementary figures and images for: Altered Thymic Function during Interferon Therapy in HCV-Infected Patients
Source: PLoS One. 2012 Apr 16;7(4):e34326. doi: 10.1371/journal.pone.0034326 (PMC3328332; doi:10.1371/journal.pone.0034326)

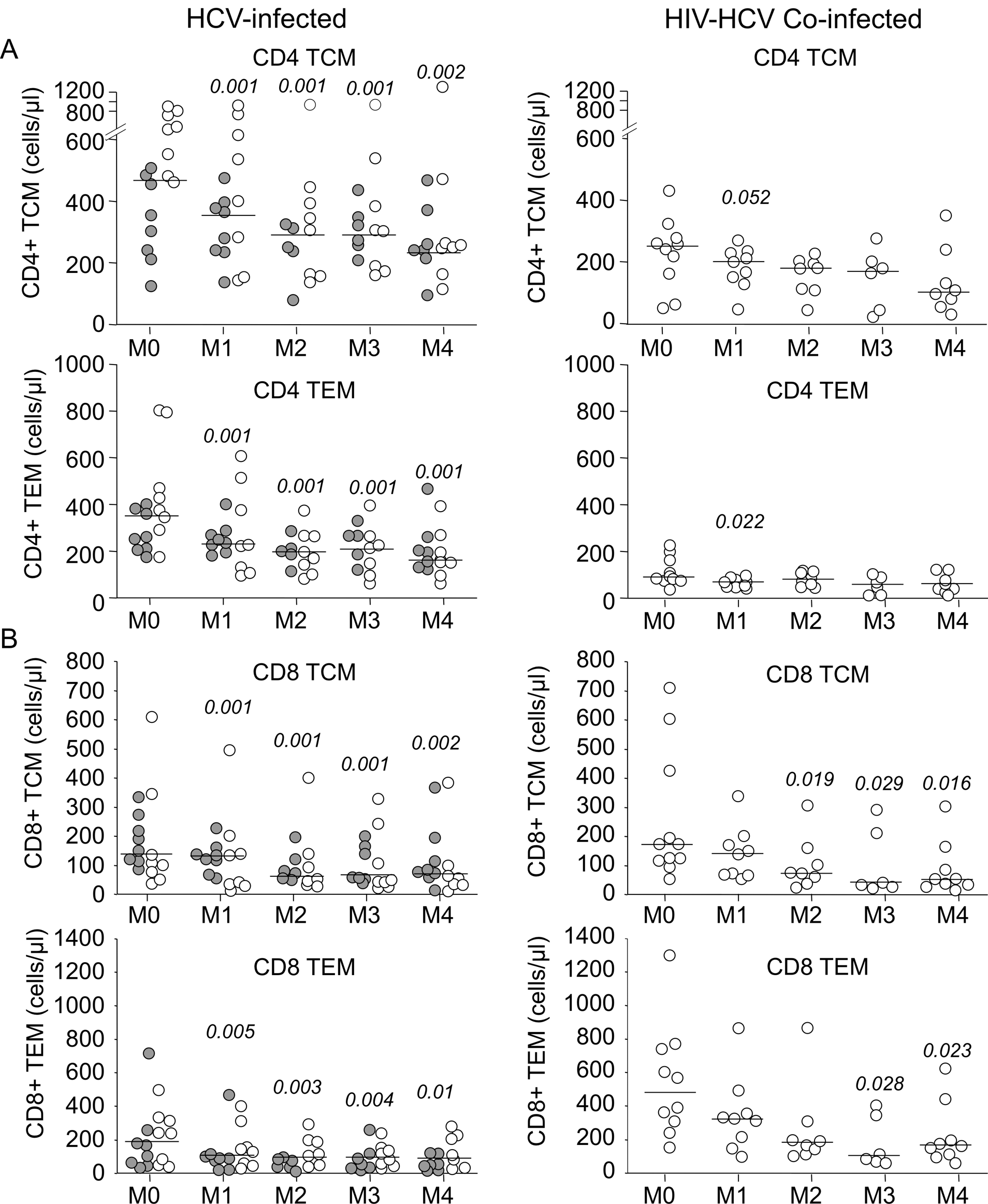

Supplement: Figure S1 — IFNα therapy leads to T-cell lymphopenia in memory compartments. Evolution of (A) CD4+ TCM (top panels) and CD4+ TEM (bottom panels) T-cell numbers, as well as (B) CD8+ TCM (top panels) and CD8+ TEM T-cell counts (bottom panels), quantified in peripheral blood cells from acutely and chronically HCV-infected (left panels white and grey symbols respectively) and HIV-HCV co-infected (right panels) patients under IFNα therapy. Horizontal bars represent median values. Statistical significance (Wilcoxon matched-pairs signed-ranks test) to baseline values (M0) are shown on top. (TIF) [file pone.0034326.s001.tif]

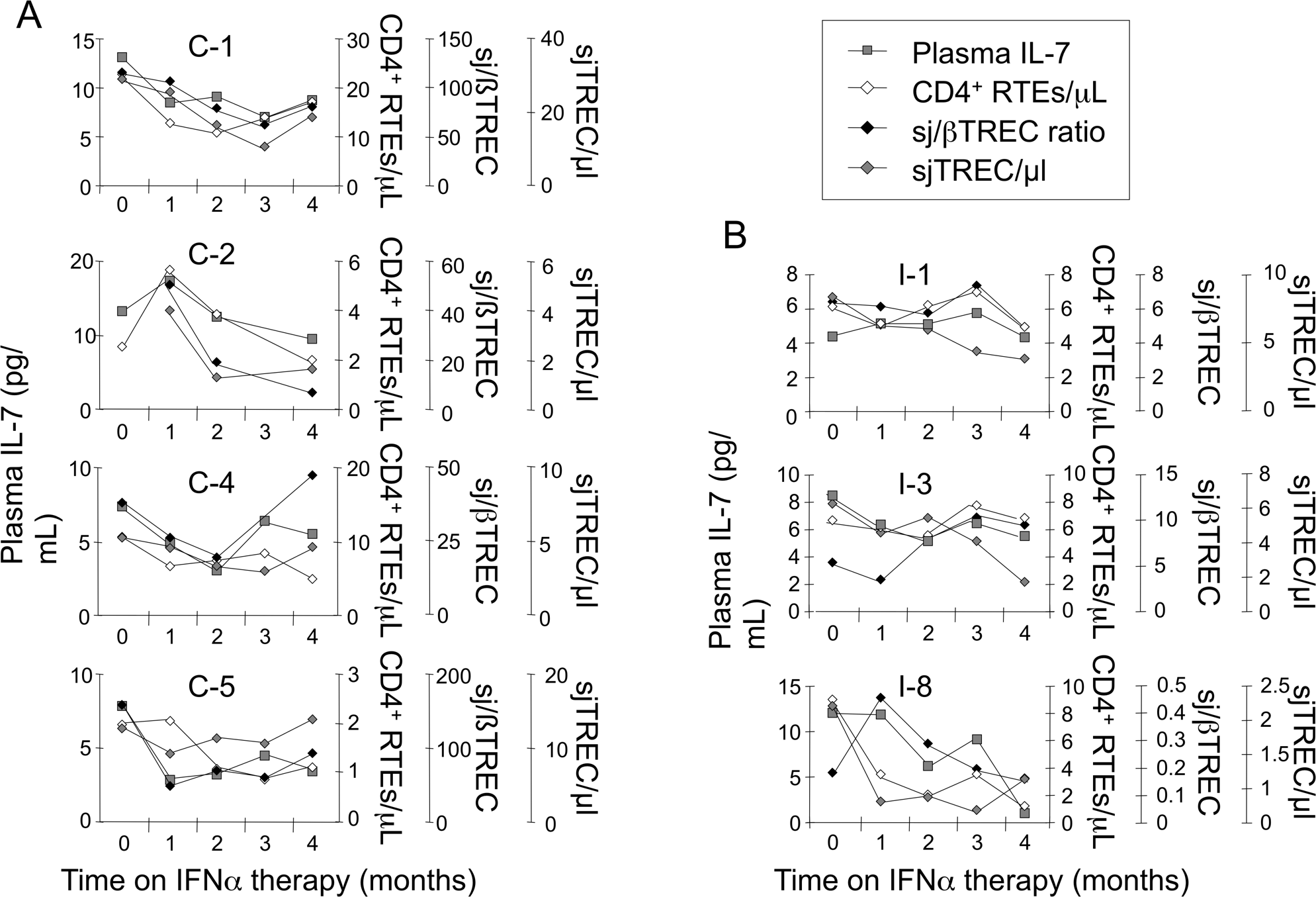

Supplement: Figure S2 — IL-7 plasma level parallels RTE concentration and thymic function. IL-7 plasma levels (grey squares), RTE (CD31Hi naïve CD4+ T-cell blood counts; open diamonds), thymic function (sj/βTREC ratio; close diamonds) and sjTREC concentrations (sjTREC/μl; grey diamonds) were longitudinally quantified in IFNα-treated HCV and HIV-HCV infected patients over a 4 month period. Representative examples of HCV-infected (A) and HIV-HCV co-infected (B) patients are shown. (TIF) [file pone.0034326.s002.tif]
